# Multiregional neuropathological changes and cellular correlates of Alzheimer’s disease

**DOI:** 10.1002/alz.090323

**Published:** 2025-01-03

**Authors:** Mariano I Gabitto

**Affiliations:** ^1^ Allen Institute for Brain Science, Seattle, WA USA

## Abstract

**Background:**

Alzheimer’s Disease is marked by the gradual aggregation of pathological proteins, Tau and beta‐amyloid, throughout various areas of the brain. The progression of these pathologies follows a consistent pattern, impacting various cellular populations as it advances through each brain region. Previously, we used Bayesian algorithms to create a continuous progression score to mathematically capture the collective aggregation of multiple pathological variables within a specific brain region. This score allowed us to discern the cellular and molecular alterations associated with the disease, offering valuable insights into the etiology of these changes within a single brain region.

**Method:**

As part of The Seattle Alzheimer’s Disease Cell Atlas (SEA‐AD, https://sea‐ad.org), we stained and quantified multiple neuropathological proteins (a‐Syn, pTDP43, pTau and beta‐amyloid) and cellular populations (neurons, astrocytes and microglia) in several brain regions (middle temporal gyrus, the middle frontal gyrus, hippocampus, and medial entorhinal cortex). Next, we profiled multiple brain regions using single nucleus RNA‐seq and a subset of donors and regions were profiled with ATAC‐seq or multiome in each SEA‐AD brain donor. We integrated these single nucleus datasets into a common latent representation, and mapped cellular types to our MTG taxonomy, adding additional cell types occurring in specific brain regions.

**Result:**

We mapped the progression of pathology using our Bayesian algorithms in each individual region. Next, we identified the order of the successive regions affected sequentially by disease. Additionally, we segmented the hippocampal formation and characterized the progression of pathology within it and linked it to synaptic connectivity across regions. Next, we used our scale of disease progression to identify the vulnerable populations in each region. After comparing populations with the known burden of pTAU and beta‐amyloid, we repeated the test to associate affected populations with the burden of each individual pathology, controlling for covariates such as sex, age and race.

**Conclusion:**

The Bayesian algorithms we developed permitted to create a continuous axis of disease progression across multiple brain regions and stage these regions given the progressive accumulation of pathology. By combining single‐nucleus experiments with detailed disease pathologies we identified a common set of affected populations in disease.

